# Collateral sensitivity increases the efficacy of a rationally designed bacteriophage combination to control *Salmonella enterica*

**DOI:** 10.1128/jvi.01476-23

**Published:** 2024-02-20

**Authors:** Luke Acton, Hannah V. Pye, Gaëtan Thilliez, Rafał Kolenda, Michaela Matthews, A. Keith Turner, Muhammad Yasir, Emma Holden, Haider Al-Khanaq, Mark Webber, Evelien M. Adriaenssens, Robert A. Kingsley

**Affiliations:** 1 Quadram Institute Biosciences, Norwich Research Park, Norwich, United Kingdom; 2 University of East Anglia, Norwich, United Kingdom; Michigan State University, East Lansing, Michigan, USA

**Keywords:** collateral sensitivity, TraDIS, LPS, BtuB, phage, antimicrobial

## Abstract

The ability of virulent bacteriophages to lyse bacteria influences bacterial evolution, fitness, and population structure. Knowledge of both host susceptibility and resistance factors is crucial for the successful application of bacteriophages as biological control agents in clinical therapy, food processing, and agriculture. In this study, we isolated 12 bacteriophages termed SPLA phage which infect the foodborne pathogen *Salmonella enterica*. To determine phage host range, a diverse collection of *Enterobacteriaceae* and *Salmonella enterica* was used and genes involved in infection by six SPLA phages were identified using *Salmonella* Typhimurium strain ST4/74. Candidate host receptors included lipopolysaccharide (LPS), cellulose, and BtuB. Lipopolysaccharide was identified as a susceptibility factor for phage SPLA1a and mutations in LPS biosynthesis genes spontaneously emerged during culture with *S*. Typhimurium. Conversely, LPS was a resistance factor for phage SPLA5b which suggested that emergence of LPS mutations in culture with SPLA1a represented collateral sensitivity to SPLA5b. We show that bacteria-phage co-culture with SPLA1a and SPLA5b was more successful in limiting the emergence of phage resistance compared to single phage co-culture. Identification of host susceptibility and resistance genes and understanding infection dynamics are critical steps in the rationale design of phage cocktails against specific bacterial pathogens.

**IMPORTANCE:**

As antibiotic resistance continues to emerge in bacterial pathogens, bacterial viruses (phage) represent a potential alternative or adjunct to antibiotics. One challenge for their implementation is the predisposition of bacteria to rapidly acquire resistance to phages. We describe a functional genomics approach to identify mechanisms of susceptibility and resistance for newly isolated phages that infect and lyse *Salmonella enterica* and use this information to identify phage combinations that exploit collateral sensitivity, thus increasing efficacy. Collateral sensitivity is a phenomenon where resistance to one class of antibiotics increases sensitivity to a second class of antibiotics. We report a functional genomics approach to rationally design a phage combination with a collateral sensitivity dynamic which resulted in increased efficacy. Considering such evolutionary trade-offs has the potential to manipulate the outcome of phage therapy in favor of resolving infection without selecting for escape mutants and is applicable to other virus-host interactions.

## INTRODUCTION

Viruses of bacteria (phages) infect and lyse bacteria and as such have the potential to replace or complement the use of antibiotics to treat bacterial infections or to control bacterial pathogens in the environment (1–3). The emergence of difficult-to-treat, antibiotic-resistant bacterial infections has renewed interest in developing products using lytic phages. Phages initiate infection of the bacterial host by specific binding to a receptor or receptors located on the cell surface of a susceptible bacteria. In Gram-negative bacteria such as *Salmonella enterica*, well-characterized receptors include outer membrane proteins, lipopolysaccharide (LPS), and flagella antigens (4). Receptor specificity of phage infection presents two challenges for the implementation of phages as antimicrobials: host specificity that limits spectrum of activity, and the potential for emergence of resistance through a single mutation. To overcome these limitations, phages are commonly pooled together to form a phage cocktail that both extends host range and limits the emergence of resistance. Selection of phages to be included in a phage cocktail should be based on a robust understanding of the bacteriophage host range, targeted receptor, and mechanisms of host resistance (5). However, mechanistic data for phage activity are often limited resulting in suboptimal cocktail design and failed applications (6, 7).

Non-typhoidal *Salmonella* (NTS) remains an important pathogen which poses a significant threat to human and livestock health and the economy. NTS is estimated to cause 93.8 million diarrheal illnesses and cause 155,000 deaths each year (8), with an associated economic cost estimated to exceed 3 billion euros in the European Union and 3.3 billion US dollars in the USA (9, 10). Most NTS infections result from the consumption of food or water that has become contaminated by feces of infected animals. Control of *Salmonella* in livestock has become increasingly challenging as resistance to commonly used veterinary antibiotics has increased. Additionally, concerns over the use of veterinary antibiotics leading to resistance to related antibiotics used to manage clinical infections in people have limited their use. Bacteriophages are a particularly promising solution to control *Salmonella* in livestock (7), but may also be appropriate in the environment of the post-slaughter food chain where antibiotics use is not possible.

We report the isolation of 12 *Salmonella enterica* bacteriophages with diverse host range and host susceptibility and resistance genes. Prediction of potential mechanisms of resistance using data from a transposon insertion mutant library functional genomic screen enabled rationale design of a combination of phages that exploited collateral sensitivity, which resulted from resistance to one phage to increase efficacy.

## MATERIALS AND METHODS

### Selection of bacterial strains

Twelve *Salmonella enterica* strains were used for the enrichment of environmental samples for bacteriophages (Table S1). Strains were of serovar Montevideo, Panama, Mbandaka, Kedougou, Infantis, Derby, Newport, Enteritidis, and Typhimurium. Eleven strains were previously isolated from food products in the UK. The remaining strain was an isogenic mutant of *Salmonella enterica* sv Typhimurium (*S*. Typhimurium) strain ST4/74 that lacked the Gifsy-1, Gifsy-2, ST46B, SopEΦ, and P4-like prophages generated previously (11). Strains used for analysis of bacteriophage host range were termed HRS (host range strain). This collection comprised 36 strains including *Salmonella enterica* from various sources*,* as well as other bacterial species isolated from food products (Table S1).

### Bacterial culture and allelic exchange

Strains were routinely cultured in Lysogenic Broth (LB) or on LB containing 1.5% agar. Where appropriate, cultures were supplemented with kanamycin (50 µg/mL) or hygromycin (75 µg/mL). Construction of mutant strains, with the exception of *S*. Typhimurium ST4/74 Δ*rfaK,* was performed by one-step inactivation using a method described previously but using the pSIM18 plasmid (12). Polymerase chain reaction (PCR) products were generated using primers listed (Table S2). The primers amplified the *aphII* gene from plasmid pKD4 and tagged it with 50 bp regions which were homologous to the target for mutagenesis at both ends. Allelic exchange was performed in *S*. Typhimurium ST4/74 containing pSIM18 (13). *S*. Typhimurium ST4/74 Δ*rfaK* was generated using gene doctoring using plasmids previously designed by Thomson et al. (14).

### Isolation of bacteriophages

All environmental samples were collected between November 2019 and February 2020. The collection included eight wastewater treatment samples, eight retail meat samples, nine lake/river samples, and six samples from drains located in a food production factory. For isolation of phage from food samples, we used supernatant from buffered peptone water (BPW) that had been inoculated with 25 g of food sample and incubated at 37°C for 18 h with shaking. Twenty-five milliliters of the BPW sample was centrifuged at 3,220 × *g* for 10 minutes and the supernatant filtered through a 0.45 µM pore size, polyethersulfone sterile syringe filter. Five milliliters was added to 40 mL of 2× LB along with 200 µL of each of the 12 previously described *Salmonella* strains cultured to mid exponential phase (OD_600nm_ of 0.6). The total volume of exponential phase bacteria added to each enrichment was 2.4 mL. The sample and culture were incubated at 37°C for 18 h. For all other samples, bacteriophages were enriched by adding 5 mL of filtered, centrifuged sample to a 12-strain enrichment broth as previously described. The resulting culture was centrifuged and filtered as previously described. New phage isolates were identified by spot assay on double agar overlay containing each of the enrichment strains to identify the host *S. enterica* strain with the greatest sensitivity to lysis as described previously (15). In cases where multiple contiguous sequences were assembled from whole-genome sequence (see below), consistent with the presence of multiple phages, a second round of isolation from phage plaques was carried out to isolate pure phage preparations.

### Phage and bacterial genomic DNA extraction and sequencing

For preparation of phage genomic DNA, high titer lysates (>10^9^ PFU/mL) were prepared by enrichment in broth cultures of the host strain that exhibited the clearest plaques and were used for nucleic acid extraction. A 1 mL aliquot of each phage was treated with 1 µL DNase (New England Biolabs, USA) at 37°C for 40 minutes. Phage virions were concentrated with 500 µL polyethylene glycol (PEG) solution (24% polyethylene glycol mw 8000, 1M NaCl) overnight at 4°C and subsequently resuspended in 200 µL of nuclease-free water following centrifugation at 21,300 × *g* for 5 minutes. Nucleic acid purification with proteinase K digestion was performed using Maxwell RSC Viral Total Nucleic Acid Purification Kit according to the manufacturer’s protocols (Promega, USA). For preparation of bacterial genomic DNA, strains were cultured at 37°C for 18 h in LB with shaking, DNA was extracted using Maxwell RSC Cultured Cells DNA Kit (Promega, USA), and Nextera XT sequencing libraries were prepared following the manufacturer’s protocols (Illumina, USA) and sequenced using the Illumina NextSeq 500 platform generating reads of 150 bases in length.

### Bioinformatic analysis

Quality control of raw, paired-end, Illumina sequencing reads carried out was using fastP (v0.23.2) with default parameters (16). Reads passing quality control were assembled *de novo* using the SPAdes-based assembler Shovill (v1.0.4) (17, 18). A Single DNA bacteriophage assembly from short-read sequence was used to infer that the phage preparation contained a single phage. In cases where multiple contiguous sequences were assembled, a further round of phage purification from plagues was carried out. For bacteriophages, sequence reads were aligned to assembled phage contigs using BWA-mem (v0.7.17.1) (19). We did not attempt to assemble reads that did not map to assembled SPLA phages. A tree based on sequence similarity of the proteome sequence was calculated using tBLASTx within the VIPTree software package and annotated using the INPHARED database (20, 21). Average nucleotide identity (ANI) comparisons were made using FastANI (v2.0) (22) . For phylogenetic reconstruction of bacterial strains of multiple genera of the order Enterobacterales, paired-end reads were assembled *de novo* using the SPAdes-based assembler Shovill (v1.0.4) and the 16S rRNA gene identified using barrnap (https://github.com/tseemann/barrnap). Multiple sequence alignments of the 16S rRNA gene was performed using MAFFT (v7.470) (23) and the alignment trimmed using trimAl (24). The resulting 1,536 base pair alignment was used to generate a maximum likelihood phylogenetic tree using IQtree 2 (v2.1.4-beta) with model selection with ModelFinder (25, 26). The selected substitution model was HKY + F + R2 with an ultrafast bootstrap value of 1,000 (27). For phylogenetic construction of *Salmonella* strains, paired-end sequences were aligned to the *S*. Typhimurium SL1344 reference genome (FQ312003) using the rapid haploid variant calling and core SNP phylogeny pipeline SNIPPY (https://github.com/tseemann/snippy) (v4.3.6) . Maximum likelihood phylogenetic trees were constructed using the multiple sequence alignment with RAxML (v8.2.12) (28) using the GTRCAT model with a bootstrapping value of 100. To identify mutations affecting phage sensitivity within phage-resistant bacteria, single-nucleotide polymorphisms (SNPs) were identified using SNIPPY (v4.3.6), comparing the ST4/74 reference genome (NC_016857.1) with sequencing reads of phage-resistant colonies.

### Bacteriophage host range, growth curve analysis, and liquid assay score (LAS)

Bacterial strains were cultured in LB for 18 h at 37°C at 200 rpm, adjusted to 1 × 10^7^ CFU/mL and 180 µL added to each well of a 96-well CytoOne microtiter plate (Starlab, UK). Bacteriophages were inoculated into wells at a multiplicity of infection (MOI) of 1. For experiments using combinations of phages, a total MOI of 1 was used. Plates were placed into FLUOstar Omega Microplate reader (BMG LABTECH, Germany) incubated at 37°C with double orbital shaking and the optical density (600 nm) was measured every 15 minutes for 18 h. For comparison of growth of *S*. Typhimurium ST4/74 wild-type and mutant strains (D*rfaL* and D*rfaK*), each biological replicate consisted of six cultures in individual wells (technical replicates). Bacterial growth curves were plotted for each biological replicate using the mean baseline corrected optical density readings of the six technical replicates. LAS was calculated using the previously published method by Xie et al. (29). Frequency of resistance to phages SPLA1a and SPLA5b in co-culture was assessed using the same method with 5 biological and 96 technical replicates. LAS was calculated using equations 1 and 2 (Supplementary Information). LAS scores were categorized as low (<33), moderate (33–66), and high (>66) susceptibility.

### Construction of transposon mutant library

To construct a Tn5 transposon mutant insertion library in *S*. Typhimurium ST4/74, transposon DNA was amplified by PCR using P-Tn5Km-01 and P-Tn5Cm-04 oligonucleotides (sequences available in Table S2) and a custom plasmid, pHPTTn5Km, as template DNA (Fig. S1). PCR products were purified using QIAquick PCR Purification Kit (Qiagen, Germany). Transposomes were prepared using 100 ng transposon DNA and EZ-Tn5 transposase (Lucigen, USA) according to the supplier’s specifications. For preparation of electrocompetent cells, a culture of *S*. Typhimurium ST4/74 was first prepared in 5 mL LB and incubated for 18 h at 37°C in a shaking incubator (200 rpm). A 500 µL aliquot of this culture was added to 50 mL 2xYT broth (1.6% tryptone, 1% yeast extract, 85.6 mM NaCl) and incubated at 37°C to OD_600_ of 0.2–0.25. Cells at the required optical density were harvested by centrifugation at 3,500 g for 10 minutes at 4°C. The resulting cells were washed three times with 10% glycerol and resuspended in 600 µL 10% glycerol. A 60 µL aliquot of electrocompetent bacterial cells were mixed with 2 µL sterile nuclease-free water, 2 µL TypeOne Restriction Inhibitor (Lucigen, USA) and 0.4 µL transposome, on ice. Following electroporation, cells were immediately resuspended in 1 mL of super optimal medium with catabolic repressor (S.O.C.) (2% tryptone, 0.5% yeast extract, 10 mM NaCl, 2.5 mM KCl, 10 mM MgCl_2,_ 10 mM MgSO_4_, and 20 mM glucose) prewarmed to 37°C and recovered at 37°C for 1.5 h. The cell suspensions in S.O.C. were then spread on LB agar supplemented with kanamycin at 50 µg/mL and these were incubated at 37°C for 16 h to select for transposon mutant colonies. Separate 10 µL and 100 µL volumes of the cell suspensions in S.O.C. were also spread on LB agar supplemented with kanamycin (50 µg/mL) for enumeration to allow an estimation of the total number of mutants obtained. Resulting colonies were harvested into LB and glycerol was added to a final concentration of 15% (vol/vol), then aliquots of this were stored at −80°C. For each experiment, a 50 µL aliquot of this stored *S*. Typhimurium ST4/74 transposon mutant library was used.

### Whole-genome functional screen using transposon-directed insertion site sequencing (TraDIS)

The transposon mutant library was challenged with six bacteriophages (SPLA1a, SPLA1b, SPLA2, SPLA5B, SPLA5c, and SPLA11) that exhibited full lytic activity against the wild-type strain *S*. Typhimurium ST4/74. The Tn5 insertion mutant library was cultured in LB at 37°C with shaking for 18 h. This culture was diluted to 1 × 10^7^ CFU/mL in 10 mL of LB containing phage added at an MOI of 10, except SPLA1a which was added an MOI of 1, and incubated at 37°C for 3 h. A buffer-only negative control containing no phage supplementation was included. A 2 mL sample of each culture was then harvested by centrifugation at 2,500 × *g* and genomic DNA was extracted as previously described. Two independent replicates were performed.

### Preparation of DNA fragments for TraDIS sequencing

Genomic DNA from the transposon mutant library with and without exposure to SPLA phages was diluted to 11.1 ng/µL and tagmented using MuSeek DNA fragment library preparation kit (ThermoFisher, USA). Fragmented DNA was purified using AMPure XP (Beckman Coulter, USA). DNA was amplified by PCR using biotinylated primers specific to the transposon and primers for the tagmented ends of DNA. PCR products were purified again using AMPure XP beads and incubated for 4 h with streptavidin beads (Dynabeads) to allow for capture of the DNA fragments with the transposon. A subsequent PCR step using barcoded sequencing primers allowed for the pooling of samples. Streptavidin beads were magnetically removed from the PCR products which were further purified and size-selected using AMPure XP beads. PCR products were quantified using Qubit 3.0 (Invitrogen, USA) and Tapestation (Agilent Technologies, USA). The PCR products were then applied to a NextSeq 500 sequencing machine fitted with a NextSeq 500/550 High Output Kit v2.5 (75 cycles) (Illumina). The nucleotide sequence reads obtained were then analyzed using the BioTraDIS (30) software suite, which aligns the sequence reads to the *Salmonella enterica* sv Typhimurium strain ST4/74 reference genome nucleotide sequence, thereby identifying the location of transposon insertions and the number of reads that match at each site. This provided an approximate number of mutants at each site. Comparison with the reference genome annotation then provides mutant information for every gene, allowing fold changes (expressed as log_2_ fold change) and statistical significance (*q*-values) to be calculated between experimental conditions for each gene using Fisher’s Exact Test as part of the BioTraDIS toolkit (30). Data where *q* < 0.05 were considered to be statistically significant.

## RESULTS

### Isolation of *Salmonella enterica* bacteriophages

To establish a diverse collection of phages capable of lysis of *S. enterica* strains, we enriched phages from wastewater, river, or food samples using a diverse mixture of *S. enterica* enrichment strains in broth culture. Plaques with distinct morphologies were picked and purified further by rounds of single plaque purification on the preferred host strain. In total, 12 phages were isolated and designated SPLA1a, SPLA1b, SPLA2, SPLA3, SPLA4, SPLA5a, SPLA5b, SPLA5c, SPLA9, SPLA10, SPLA11, and SPLA12 (Table 1). Illumina short-read sequencing of nucleic acid prepared from each phage lysate assembled into a single contiguous sequence for each phage, with contigs ranging in size from 40,585 bp to 240,593 bp. Except for SPLA1b, whole-genome sequence reads of the *S. enterica* strains used to enrich the phage did not align to the assembled phage genomes, indicating that these were not prophage-derived from the enrichment strains. Sequence reads corresponding to a prophage in *S*. Typhimurium strain B8 C7 aligned to the full length of the SPLA1b-assembled sequence, indicating that this phage was likely activated from the enrichment strain.

**TABLE 1:** Summary of SPLA bacteriophage characteristics

	Isolation host serovar	Source	Genome assembly (bp)	Predicted family	Predicted genus	Accession
SPLA1a	*S*. Typhimurium	Food	240,348	Unclassified	*Seoulvirus*	OR413578
SPLA1b	*S*. Typhimurium	Food	39,459	Unclassified	*Lederbergvirus*	OR413579
SPLA2	*S*. Newport	Food	53,075	Unclassified	*Rosemountvirus*	OR413580
SPLA3	*S*. Mbandaka	River	240,593	Unclassified	*Seoulvirus*	OR413581
SPLA4	S. Mbandaka	Factory drain	39,540	*Autographiviridae*	*Berlinvirus*	OR413582
SPLA5a	*S*. Montevideo	Wastewater influent	151208	Unclassified	*Seunavirus*	OR413583
SPLA5b	*S*. Kedougou	Wastewater influent	107,599	*Demerecviridae*	*Tequintavirus*	OR413584
SPLA5c	*S*. Infantis	Wastewater influent	239,099	Unclassified	*Seoulvirus*	OR413585
SPLA9	*S*. Montevideo	Wastewater influent	51,694	Unclassified	*Rosemountvirus*	OR413586
SPLA10	*S*. Montevideo	Wastewater influent	210,197	Unclassified	*Phikzvirus*	OR413575
SPLA11	*S*. Panama	Wastewater influent	52,451	Unclassified	*Rosemountvirus*	OR413576
SPLA12	*S*. Infantis	Wastewater influent	51,974	Unclassified	*Rosemountvirus*	OR413577

### SPLA phages are diverse and represent seven distinct phage lineages

**Fig 1:**
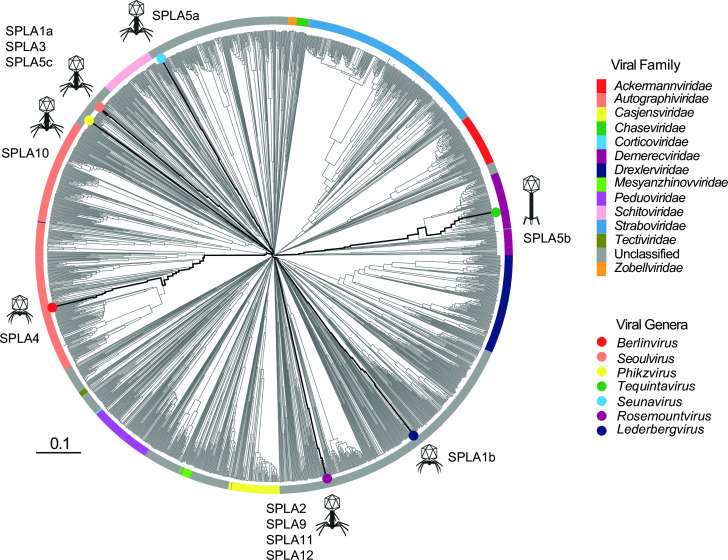
Relationship of SPLA phages proteome in the context of known diversity of viral proteomes from viral families known to infect Gammaproteobacteria. The dendrogram was generated using the proteome data with VipTree software and viral family was predicted using INPHARED. SPLA *Salmonella* phage isolates with circles colored by the viral genera *Berlinvirus* (red), *Seoulvirus* (orange), *Phikzvirus* (yellow), *Tequintavirus* (green), *Seunavirus* (light blue), *Rosemountvirus* (purple), and *Lederbergvirus* (dark blue). Icons indicate predicted phage morphology.

To investigate the diversity and relationship to known phage genome sequences in available databases, SPLA phages were placed in a phylogenetic context based on sequence similarity of their predicted proteome sequence. Phylogenetic reconstruction using variation in the predicted proteome of SPLA phages with phage sequences in the virus-host database indicated that all SPLA phages clustered with phages known to infect Gammaproteobacteria (Fig. S2). Therefore, to improve clarity, the analysis was repeated with only phages of Gammaproteobacteria (Fig. 1). The SPLA phages were identified as members of the class *Caudoviricetes* and were present in clusters on seven deeply rooted lineages that corresponded to the genera *Berlinvirus*, *Seoulvirus*, *Phikzvirus*, *Tequintavirus*, *Seunavirus*, *Rosemountvirus,* and *Lederbergvirus*. SPLA1a, SPLA3, and SPLA5b were predicted to be myophages of the genus *Seoulvirus* (31). SPLA1a, SPLA3, and SPLA5b had an ANI greater than 98.4% and should be considered strains of the same species. SPLA2, SPLA9, SPLA11, and SPLA12 were assigned to the genus *Rosemountvirus* and exhibited greater than 95.6% ANI. Phages SPLA4 and SPLA5b were the only phages which are currently assigned to established viral families, *Autographiviridae* and *Demerecviridae,* respectively, according to the ICTV database (accessed December 2022).

### SPLA phages are highly specific to *Salmonella* serovars

**Fig 2:**
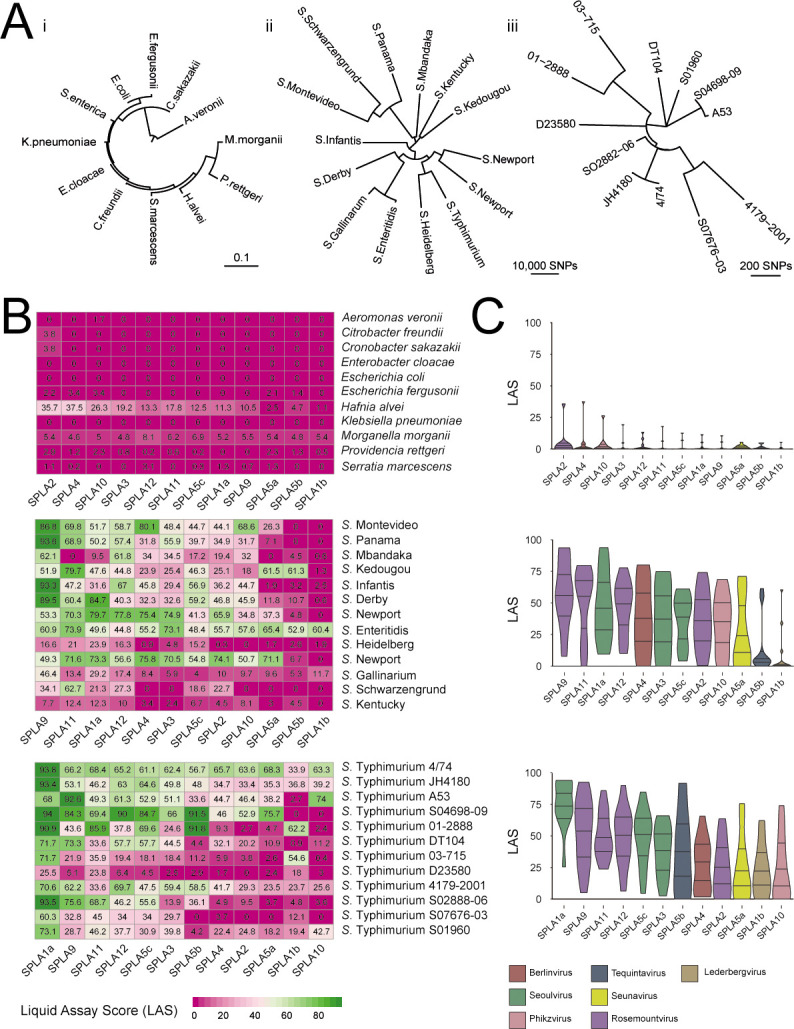
Host range of SPLA phages for strains of diverse gammaproteobacterial species and strains of *S. enterica*. (**A**) Maximum likelihood phylogenetic trees showing the relationship and genetic distance of bacterial strains used for determination of host range based on SNPs in the 16S rRNA sequence of 12 strains of proteobacterial species (i) and core genome of 13 serovars (ii) and 12 strains of serovar Typhimurium (iii). (**B**) Heat map showing LAS ranging from 0 (no change in growth) to 100 (no growth) of each phage host bacterial strain combination of 12 strains of proteobacterial species (top) and core genome of 13 serovars (center) and 12 strains of serovar Typhimurium (bottom). (**C**) Violin plots of LAS scores for 12 strains of proteobacterial species (top) and core genome of 13 serovars (center) and 12 strains of serovar Typhimurium (bottom) in the presence of each SPLA phage.

To determine the host range of the SPLA phages, susceptibility of 36 bacterial strains of 11 diverse gammaproteobacterial species were tested (Fig. 2A). These included a representative strain from each of the 10 species of Enterobacterales, a strain of Aeromonaceae, and 25 strains of *S. enterica* comprising 14 different serovars (Fig. 2A). Strains of species other than *S. enterica* exhibited low susceptibility to SPLA phages indicated by the LAS (0–10) except for moderate susceptibility of a *Hafnia alvei* strain to several SPLA phages, particularly to SPLA2 and SPLA4 (Fig. 2B). Susceptibility of *S. enterica* strains to SPLA phages varied markedly, with all phages exhibiting moderate (33–66 LAS) to high virulence (>66 LAS) to one or more strains of all 14 serovars tested. The taxonomically diverse phages SPLA9, SPLA11, and SPLA1a exhibited the broadest host range, displaying at least moderate virulence for greater than 9 of the 14 serovars tested (Fig. 2B and C). Nonetheless, all except SPLA5a, SPLA5b, and SPLA1b had at least moderate virulence against at least six of the serovars tested (Fig. 2B and C). Although *S*. Typhimurium strains were considerably more closely related to one another compared with strains of distinct serovars, SPLA phages exhibited similar variability in virulence. Phage SPLA1a was particularly virulent for a broad range of *S*. Typhimurium strains and SPLA9 and SPLA11 were also moderately virulent for at least 11 of the 12 strains tested. We also observed that even very closely related *Salmonella* strains exhibited diversity in sensitivity to SPLA phages. For example, *S*. Typhimurium strains S04698-09 and A53 were both part of the monophasic *S*. Typhimurium ST34 epidemic clade that emerged in the last three decades (32, 33), yet these strains exhibited distinct differences in susceptibility to at least five SPLA phages. Furthermore, a *S*. Typhimurium strain of ST4/74 that was genetically modified to remove prophage elements from its genome exhibited a moderate decrease in sensitivity to several SPLA phages.

### Identification of SPLA phage host susceptibility and resistance genes using a functional genomics screen

To identify bacterial genes affecting the virulence of infecting SPLA phages, we constructed a Tn5 transposon insertion mutant library in *S*. Typhimurium strain ST4/74 comprising over 600,000 unique insertion sites corresponding to one insertion every seven base pairs of the genome sequence, on average. In a preliminary screen to determine susceptibility of the wild-type strain of the transposon mutant library to the SPLA phages, we found that SPLA1a, SPLA1b, SPLA2, SPLA5b, SPLA5c, and SPLA11 had a LAS >50, indicating moderate to high virulence of the phages in question.

**Fig 3:**
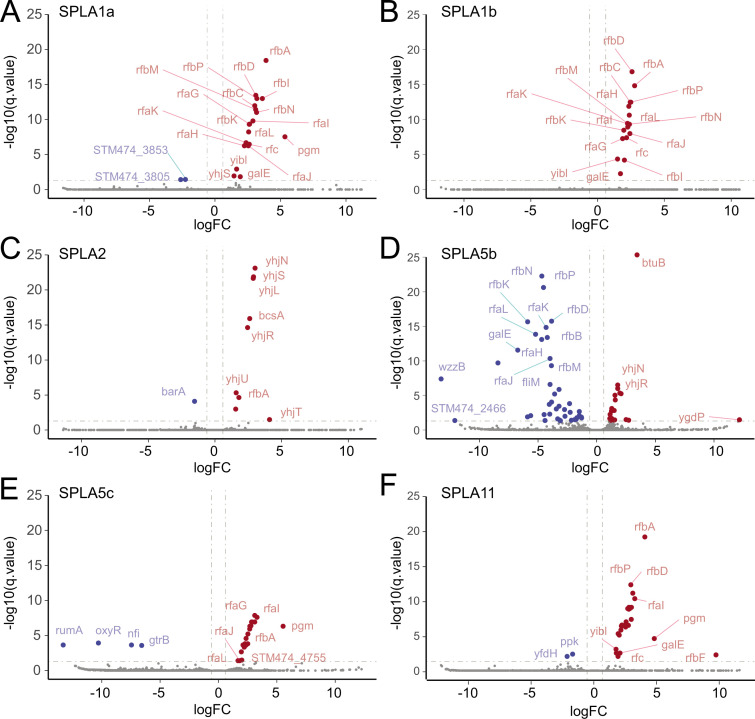
Changes in the transposon insertions within genes in response to predation by six SPLA phages. Log2 fold change in insertions (x-axis) is plotted against -log_10_(q-value) statistical significance (y-axis) of difference in the number of insertions in each gene in cultures of S. Typhimurium treated with phage or untreated. Each graph shows gene hits for treatment with phage SPLA1a (**A**), SPLA1b (**B**), SPLA2 (**C**), SPLA5b (**D**), SPLA5c (**E**), and SPLA11 (**F**). Genes with significantly fewer insertions (blue points) or greater insertions (red points) are indicated. Selected genes are labeled with lines indicating the relevant data point.

TraDIS was used to identify mutants whose abundance changed following cultures with each of these six bacteriophages compared to a control without phage treatment. Genes that exhibited an increase in frequency of tranposon insertions were termed susceptibility genes since their inactivation improved bacterial survival. Most of the susceptibility genes identified were genes which encoded proteins or were involved in the biosynthesis of macromolecules present on the outer surface of the bacterium (Fig. 3). For example, insertional inactivation of *btuB,* that encodes a vitamin B12 (cobalamin) transporter, was identified as a susceptibility gene for infection by phage SPLA5b (Fig. 3D), while genes such as those in the *rfa* and *rfb* loci involved in the biosynthesis of LPS were identified for SPLA1a, SPLA1b, and SPLA5c (Fig. 3A, B, and E). Likewise, inactivation of genes including *yhjU*, *yhjL*, *yhjN*, *yhjQ*, *yhjR,* and *yhjS,* involved in the biosynthesis of cellulose, reduced susceptibility to SPLA2 (Fig. 3C).

In contrast, genes in which insertional inactivation mutants decreased in frequency following selection by SPLA phages were determined to provide resistance to bacteriophages. Putative resistance genes included two regulatory genes *barA* (SPLA2) and *oxyR* (SPLA5c), *rumA* involved in methylation of uracil in 23S ribosomal RNA (SPLA5c), *nfi* encoding an endonuclease (SPLA5c), and *gtrB* involved in glycosylation of LPS (SPLA5c and SPLA11). Notably, while genes involved in synthesis of LPS were susceptibility genes for SPLA1a, SPLA1b, and SPLA5c, these genes were resistance genes for infection by SPLA5b. Notably, while insertional inactivation of genes coding for synthesis of LPS increased susceptibility to phages SPLA1a, SPLA1b, and SPLA5c, it reduced susceptibility to phage SPLA5b.

### Closely related seoulviruses SPLA1a and SPLA5c differ in virulence due to sensitivity to O-antigen glycosylation by GtrB

**Fig 4:**
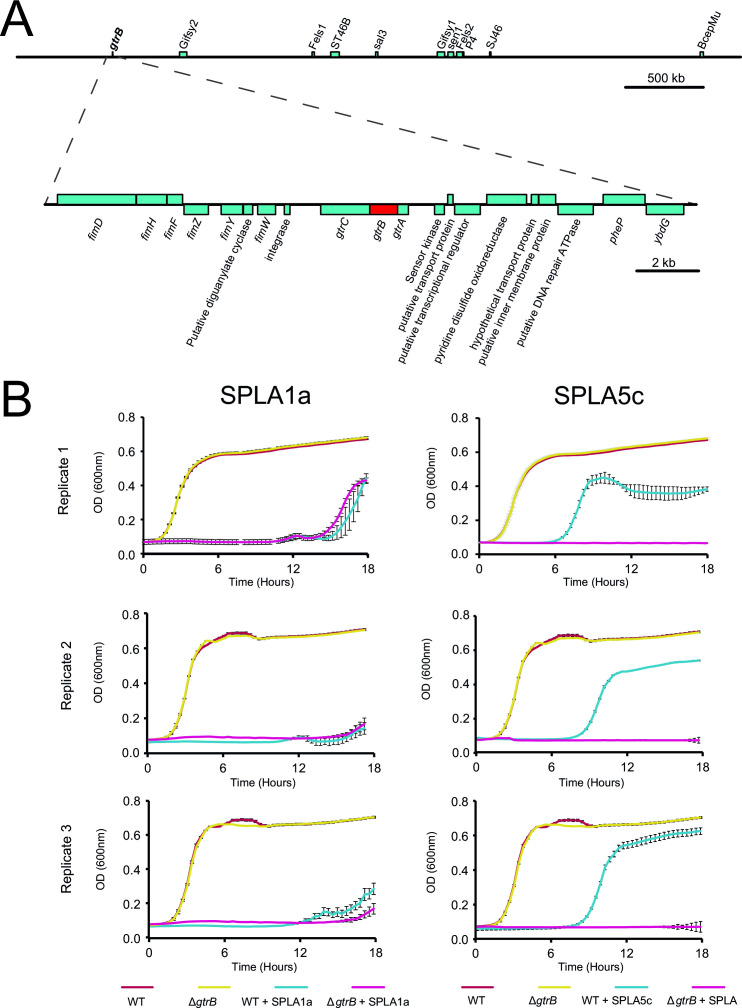
The role of *gtrB* in phage resistance to SPLA5c. (**A**) Genomic context of *gtrABC* genes in *Salmonella* Typhimurium ST4/74 with *gtrB* (red bar) and neighboring genes (teal) indicated on forward and reverse strands above and below horizontal line. (**B**) Growth of *S*. Typhimurium strain 4/74 and an otherwise isogenic strain in which the *gtrB* gene had been replaced by the *aphII* gene (Δ*gtrB*) in the presence or absence of SPLA1a (left graphs) and SPLA5c (right graphs). Data from three biological replicates, each showing the standard error of the mean from six technical replicates are shown.

SPLA1a and SPLA5c exhibited distinct virulence from each other to a range of strains of various *S. enterica* serovars, and to a range of strains of serovar *S*. Typhimurium (Fig. 2), despite sharing 97% nucleotide sequence identity across their whole genome. For example, SPLA1a exhibited high virulence (LAS = 94) while SPLA5c exhibited moderate virulence (LAS = 61) for *S*. Typhimurium strain ST4/74. A key functional difference between SPLA1a and SPLA5c was that virulence of SPLA5c was sensitive to the presence of the *Salmonella*-encoded *gtrB* gene (Fig. 4A), encoding a glucosyltransferase. The glucosyltransferase gene was not prophage encoded which is common for genes within this family (34). A *S*. Typhimurium strain ST4/74 in which the *gtrB* gene was replaced by an *aphII* gene resulted in no bacterial growth in the presence of SPLA5c, indicating comparable virulence to SPLA1a (Fig. 4B). To investigate differences in nucleotide sequence of SPLA1a and SPLA5c that may account for the distinct sensitivity of each phage to the *gtrB* phage resistance gene, the genomes were aligned and regional variation in nucleotide sequence identity and insertions or deletion were identified. SPLA1a had two insertions relative to SPLA5c affecting a gene of unknown function and a region encoding a putative virion structural protein. SPLA5c had three insertions affecting a gene encoding a putative RNA polymerase subunit, the terminase large subunit, and a protein of unknown function (Fig. S3). There was also a large region of approximately 10 kb with greater sequence divergence affecting 17 genes that mostly encoded proteins of unknown function, but also virion structural proteins and a putative tail fiber protein.

### Functional genomics predicts collateral sensitivity of resistant escape mutants and enables rational design of phage combinations with increased efficacy

**Fig 5:**
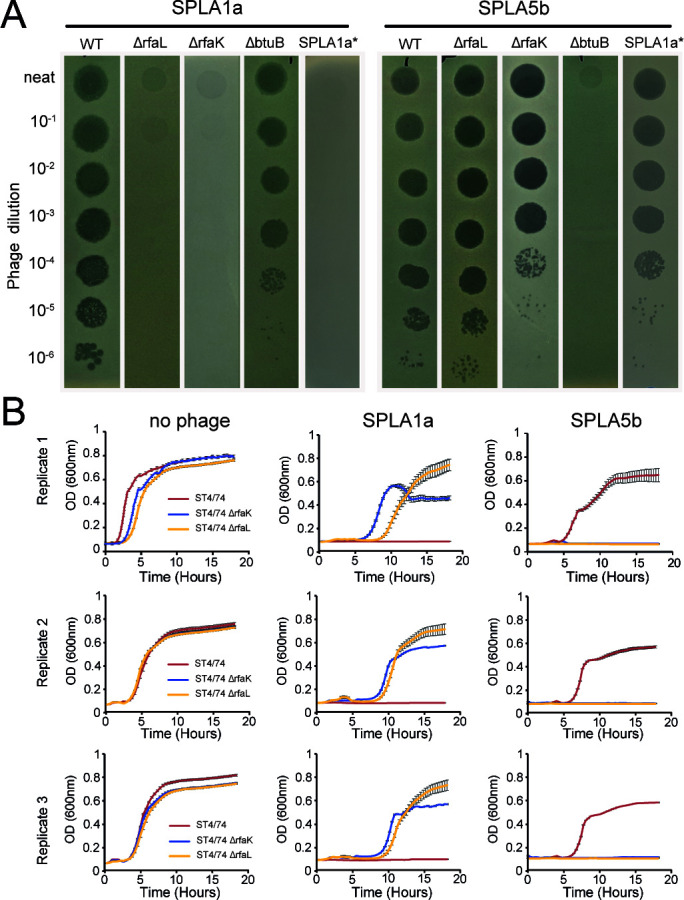
Inactivation of genes in *S*. Typhimurium strain ST4/74 that confer resistance to SPLA1a results in increased susceptibility to SPLA5b phage. (**A**) Plaque assays using SPLA1a (left) and SPLA5b (right) against LPS and BtuB Mutants. (**B**) Growth in broth culture of *S*. Typhimurium strain ST4/74 (red) or an otherwise isogenic strain in which the *rfaK* or *rfaL* genes were deleted [Δ*rfaK* (blue), Δ*rfaL* (yellow)] when treated with no phages (left graphs), SPLA1a (middle graphs), and SPLA5b (right graphs). Data from three biological replicates, each showing the standard error of the mean from six technical replicates, are shown.

Collateral sensitivity is an evolutionary trade-off where resistance to one antimicrobial agent results in increased sensitivity to another (35). We observed that LPS biosynthesis genes played contrasting roles as resistance genes or sensitivity genes during infection with phage SPLA1a and SPLA5b, respectively. To investigate the differential role of LPS in virulence of SPLA1a and SPLA5b phages, their virulence was determined for otherwise isogenic *S*. Typhimurium ST4/74 strains in which *rfaL* or *btuB* were deleted. Both SPLA1a and SPLA5b were able to form plaques on wild-type *Salmonella* Typhimurium ST4/74, although plaques formed by SPLA5b were more turbid than those formed by SPLA1a, consistent with a lower virulence of the former (Fig. 5A). Deletion of *rfaL* resulted in complete resistance to SPLA1a, as indicated by the lack of plaques in overlay assays, consistent with O-antigen being the receptor for this phage. In contrast, SPLA5b was still able to infect the *rfaL* mutant, as expected from the TraDIS data, but plaques were markedly less turbid, suggesting an increase in phage sensitivity. Deletion of *rfaK*, that encodes a hexose transferase involved in LPS synthesis, resulted in an unexpected decrease in plaquing efficiency, suggesting that mutations affecting different LPS biosynthesis functions have variable effects on phage plaquing. Loss of the vitamin B12 transporter, by disruption of the *btuB* gene, resulted in resistance to SPLA5b as indicated by the lack of plaques, but no effect on SPLA1a infection (Fig. 5). This was consistent with BtuB being the receptor for SPLA5b.

To further determine if LPS modification resulted in increased susceptibility to SPLA5b, the virulence of SPLA phages on ST4/74 strain lacking the *rfaK* gene (Δ*rfaK*) or *rfaL* (Δ*rfaL*) was determined. No growth of wild-type ST4/74 was observed in the presence of SPLA1a, but strains with a deletion in *rfaK* or *rfaL* resulted in the ability to grow under SPLA1a selection. Notably, *rfaL* mutants were able to consistently grow to a higher optical density than *rfaK* mutants in the presence of SPLA1a, which may be due to the *rfaL* mutant affecting LPS structure in a way that affected the infection process more completely. In contrast, growth was observed when wild-type ST4/74 was infected with SPLA5b, despite the ability of this phage to produce clear plaques (Fig. 5A), with the phage causing a delay in the time to initial observation of growth compared to non-phage-treated conditions. However, loss of *rfaK* or *rfaL* resulted in increased phage infection by SPLA5b, compared to the wild-type (Fig. 5B). In particular, SPLA5b was able to plaque efficiently but did not exhibit strong virulence in liquid assay against the wild-type strain. This could be explained by differences in conditions between the assays which have been described previously (29).

**Fig 6:**
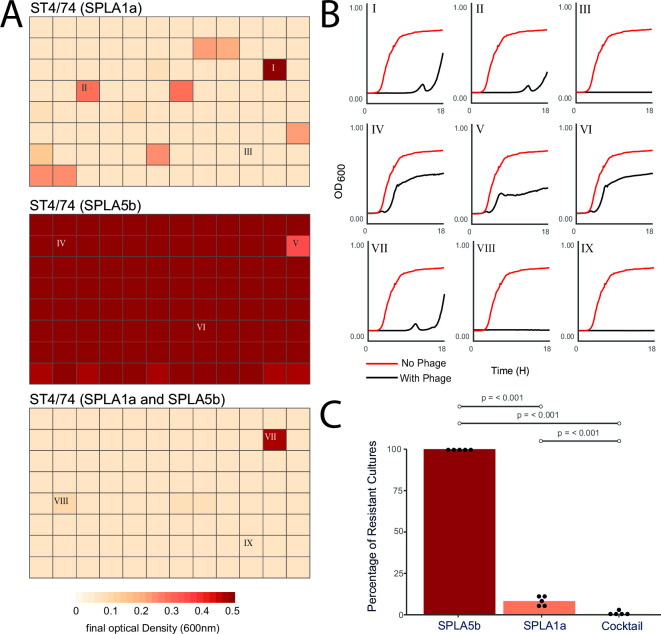
Growth of *Salmonella* Typhimurium ST4/74 in the presence of SPLA1a and SPLA5b in single and co-culture. The end point optical density at 600 nm (OD_600 nm_) after 18 h culture of *S.* Typhimurium strain ST4/74 in the presence of phage SPLA1a (top), phage SPLA5b (middle), or SPLA1a and SPLA5b (bottom) (**A**). Example growth curves of *Salmonella* Typhimurium ST4/74 infected by SPLA1a (I–III), SPLA5b (IV–VI), and SPLA1a and SPLA5b (VII–IX) for which roman numerals correspond to culture end points labeled previously (**B**). Percentage of resistant cultures for SPLA1a and SPLA5b, and combination treatment across five biological replicates. Statistical comparisons between phage combinations using a Student’s *t*-test are indicated (*P* < 0.001).

Since mutations in the same receptor were confirmed to play contrasting roles in infection by SPLA1a and SPLA5b, it raised the possibility that naturally occurring resistance to SPLA1a may result in increased sensitivity to SPLA5b. Therefore, collateral sensitivity may improve the efficacy of these phages in combination treatment. In cultures of *S*. Typhimurium ST4/74 infected with SPLA1a, approximately 10% (10/96) resulted in the emergence of naturally occurring resistance within 18 h of incubation at 37°C (Fig. 6). Whole-genome sequence of a resistant strain (4/74 SPLA1a*) revealed a single base insertion in the *pgm* gene that resulted in a frame shift predicted to truncate this gene (Fig. S4). The *pgm* gene, encoding phosphoglucomutase, was also identified as a host susceptibility gene for SPLA1a phage in the transposon mutant library screen (Fig. 3A). In contrast to SPLA1a, upon infection of *S*. Typhimurium ST4/74 with SPLA5b, host resistance emerged in 100% (96/96) of cultures, although growth was delayed, and the final optical density was lower than uninfected controls (Fig. 6). Consistent with the idea that resistance to SPLA1a results in collateral sensitivity to SPLA5b, both phages were used in combination and growth was only observed in 1/96 of cultures, an approximate 10-fold reduction in phage resistance compared to SPLA1a alone. This reduction was consistently observed across five biological replicates.

**Fig 7:**
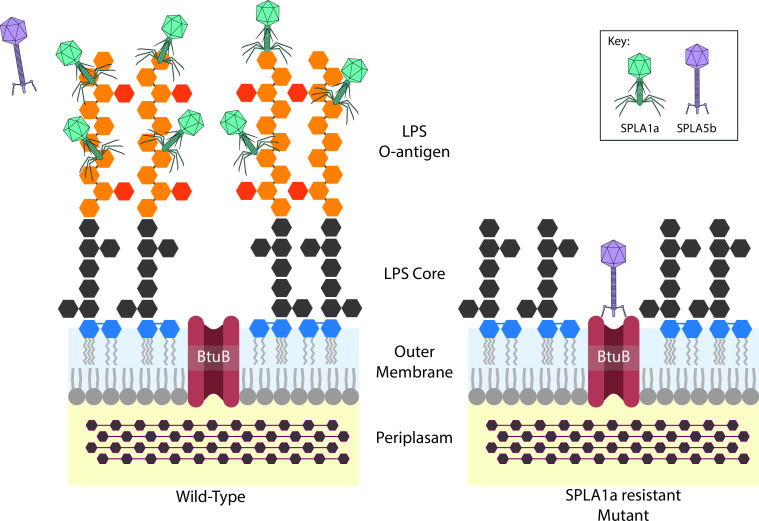
Schematic of predicted collateral sensitivity mechanism observed in combination treatment with SPLA1a and SPLA5b. Wild-type strains (left) are highly susceptible to SPLA1a, but less susceptible to SPLA5b due to receptor masking. SPLA1a-insensitive mutant (right) is no longer infected by SPLA1a, due to lack of O-antigen receptor, but has increased susceptibility to SPLA5b infection due to loss of receptor masking.

Based on the results of our liquid growth assays, we propose that resistance to SPLA1a due to mutations affecting production of LPS results in a collateral sensitivity dynamic, increasing sensitivity to SPLA5b. A working hypothesis that is consistent with our observations is that wild-type *S*. Typhimurium ST4/74 strains that elaborate LPS with long chain O-antigen on their surface mask the BtuB receptor used by SPLA5b for initiation of infection. Mutations in LPS biosynthesis, such as Δ*rfaL*, lead to resistance to SPLA1a but provide greater access to the BtuB receptor and increased sensitivity to SPLA5b (Fig. 7).

## DISCUSSION

Phages show potential as antimicrobials to control bacterial pathogens in the environment or to replace or augment therapies to treat infections. Two of the main technological challenges to the development of phage-based products is their specificity and the rapid emergence of resistance by target bacteria. Although strain or species specificity is a potential advantage, as it is preferable that bystander bacteria are not affected during application of the bacteriophage, host specificity can be a challenge for the development of broadly applicable products. A common solution is to formulate a cocktail of several phages with diverse host ranges and receptors. This approach may also contribute to decreasing the likelihood of the emergence of resistance since multiple mutations may be required to produce resistance to the cocktail of phages. Previous studies have shown that phage cocktails are effective against many pathogens including *Enterococcus*, *Salmonella,* and *Campylobacter* (36–38). In this study, we devised a functional genomics approach to direct the formulation of combinations of newly isolated phages, using a transposon mutant screen in conjunction with TraDIS to identify genes that either increased sensitivity, susceptibility genes, and genes that decreased sensitivity to genes, resistance genes.

The formulation of optimal phage cocktails is likely to depend on combining phylogenetically diverse phages, as related phages are likely to use identical or similar bacterial encoded receptors to initiate infection. Furthermore, their susceptibility to resistance mechanisms encoded by the target bacteria is likely to be shared. We isolated 12 phages that represented seven different viral genera, based on whole-genome sequence and proteome comparison. All phages had limited virulence for strains of species other than *Salmonella enterica*, signifying their potential suitability for applications where specificity is important. Notably, the greatest off-target virulence was observed for a strain of *Hafnia alvei*, a species that is relatively distantly related to *Salmonella enterica*, while all phages were completely avirulent for an *Escherichia coli* strain that was more closely related to *Salmonella enterica*. However, it should be noted that only a single *E.coli* strain was tested, and a host range of bacteriophages infecting *E. coli* has previously been shown to be highly variable (39).

Considerable variation in virulence of all phages was observed for representative strains of 14 serovars of *Salmonella enterica*, highlighting the importance of combinations of phages to ensure broad activity. One or more phages showed moderate to high virulence to serovars Typhimurium, Newport, and Derby that together account for 89% of pig isolates in 2020 and serovars Kedougou, Montevideo, Mbandaka, Newport, and Enteritidis that together accounted for 46% of poultry isolates in 2020 , supporting their potential for development as antimicrobials in the food chain. A similar level of variation in virulence of SPLA phages for 11 strains of *S*. Typhimurium to that for representative strains of diverse serovars was observed. This was perhaps surprising since *S*. Typhimurium exhibits less than 0.1% nucleotide sequence divergence in their genome (40), compared with up to 2% nucleotide sequence divergence for strains of *S. enterica* (32). *S*. Typhimurium accounts for almost one-quarter of all *Salmonella* isolated from humans and 78% of pig isolates in 2020 in the UK (41); this serovar is of particular interest for interventions. *S*. Typhimurium is particularly widespread in nature, being in the top three serovars isolated from multiple species of livestock and wild animals, and is also relatively genetically diverse compared to other serovars with multiple lineages exhibiting broad host range or host adaptation (42). This level of diversity has not been observed for other serovars and it remains to be determined if a similar level of variation in sensitivity to phage also occurs in strains of serovars other than Typhimurium.

Phage SPLA1a was particularly virulent for multiple *S*. Typhimurium strains, exhibiting high virulence for all but strain D23580. Strain D23580 is from a clade of sequence type 313 associated with invasive disease in sub-Saharan Africa and is rarely associated with infection outside of this geographical location (43, 44). Other SPLA phages also exhibited low virulence or were unable to infect strain D23580. This strain has a unique prophage repertoire including BTP-1 and BTP-5 (45, 46). BTP-1 harbors the phage resistance gene *bstA* which confers population-level resistance to bacteriophages via abortive infection and may explain the relative phage resistance observed in this strain. SPLA1a was also the third most virulent SPLA phage for strains from diverse serovars of *S. enterica* and is therefore a potential key component of phage cocktails. Despite the relatively high virulence of SPLA1a, approximately 10% of *S*. Typhimurium ST4/74 cultures infected with the phage exhibited some growth around 18 h later.

Infection by bacteriophages is a complex process with host bacteria encoding many genes affecting infection (47). Transposon insertion mutant library screens have been used to identify host genes involved in infection by model phages such as for T4 (48), λ (49) T2, T6, and T7 phages (50). Additionally, it has been used to identify capsule polysaccharides as the receptor for novel phage, RAD2, in *Klebsiella* and a Vi phage of *S*. Typhi (51). However, it is not common practice to identify host resistance and susceptibility genes during the development of phage cocktails (52). Three of five lytic phages investigated here, SPLA1a, SPLA5c, and SPLA11, likely recognize the LPS as the primary receptor, while SPLA2 and SPLA5b recognize cellulose and BtuB, respectively. These susceptibility genes are candidate genes that may be inactivated in strains that become resistant on exposure to phages. A common mechanism of defense against O-antigen targeting phages is the deletion of LPS biosynthesis genes or epigenetic control of O-antigen chain length (53, 54). We also observed a mutation in the *pgm* gene of a resistant strain of *S*. Typhimurium that emerged during culture with SPLA1a phages. No previously described specific resistance mechanisms were identified. However, genes encoding regulators OxyR and BarA that may repress expression of a phage receptor in the test culture condition were identified as candidate resistance genes for SPLA5c and SPLA2, respectively. Also, the *nfi* gene that is predicted to encode an endonuclease involved in DNA repair (55) may play a role in phage resistance to SPLA5c, and genes that modified the O-antigen receptor by glycosylation, thereby preventing access to the O-antigen receptor, were identified. These genes are of potential interest as molecular markers of resistance that could be used to predict the outcome of phage therapy.

Of particular interest for rational design of phage cocktails was the observation that some genes were susceptibility genes for one group of phages and a resistance gene for a second phage. This was the case of LPS biosynthesis genes that were susceptibility genes for SPLA1a, SPLA5c, and SPLA11, and resistance genes for SPLA5b. Mutations in susceptibility genes identified in our transposon insertion mutant library are candidate genes that may be inactivated in strains that become resistant on exposure to phages. To exploit this resistance mutation, we tested the efficacy of a mixture of SPLA1a with SPLA5b and found that addition of SPLA5b increased efficacy by 10-fold. Previous studies describing the formulation of phage cocktails were likely to have also exploited collateral sensitivity (56, 57) and at least one study specifically including phage that retained virulence to escape mutants (58).

Perhaps, the greatest challenge for successful implementation of phage therapy are concerns of the rapid acquisition of phage resistance and treatment failure. Our data support the idea that the use of functional genomic screens is effective in identifying phages that target phage-resistant escape mutants of additional phages facilitating the design of cocktails with improved efficacy. Additionally, identification of the genetic determinants of phage susceptibility can be used to help predict which strains a phage or phage cocktail can infect, which is likely to be important for successful implementation of phage therapy.

## Data Availability

The genome sequence of SPLA phages are publicly available within NCBI with individual accessions listed in Table 1. Sequence reads of bacteria are deposited within the Sequence Read Archive (SRA) with accessions listed in Table S1. TraDIS sequencing reads are deposited within SRA and available under the project PRJNA1004457. Bacteriophage sequencing reads are available under the project code PRJNA1031836.

## SUPPLEMENTAL MATERIAL

Supplemental material jvi.01476-23-s0001.docx Supplemental equations and figures; legends for supplemental tables.

Table S1 jvi.01476-23-s0002.xlsx Bacterial strain collection used in the study for the isolation of bacteriophages and the investigation of bacteriophage host range.

Table S2 jvi.01476-23-s0003.xlsx Primer sequences used in the study.

Table S3 jvi.01476-23-s0004.xlsx Log fold changes and statistical significance of transposon insertions within genes of *Salmonella* Typhimurium strain ST4/74 following exposure to six SPLA bacteriophages.

ASM does not own the copyrights to Supplemental Material that may be linked to, or accessed through, an article. The authors have granted ASM a non-exclusive, world-wide license to publish the Supplemental Material files. Please contact the corresponding author directly for reuse.
