# Supplementary material for: Collateral sensitivity increases the efficacy of a rationally designed bacteriophage combination to control Salmonella enterica
Source: J Virol. 2024 Feb 20;98(3):e01476-23. doi: 10.1128/jvi.01476-23 (PMC10949491; doi:10.1128/jvi.01476-23)
Supplement: Supplemental material — Supplemental equations and figures; legends for supplemental tables. [file jvi.01476-23-s0001.docx]

**Collateral sensitivity increases the efficacy of a rationally designed bacteriophage combination to control *Salmonella enterica***

Luke Acton^1^, Hannah Pye^1^, Gaëtan Thilliez^1^, Rafał Kolenda^1^, Michaela Matthews^1^, A. Keith Turner^1^, Muhammad Yasir^1^, Emma Holden^1^, Haider Al-Khanaq^1^, Mark Webber^1,2^, Evelien M Adriaenssens^1^, Robert A Kingsley^1,2^

^1^ Quadram Institute Biosciences, Norwich Research Park, Norwich, NR4 7UQ, UK.

^2^ University of East Anglia, Norwich, NR4 7TJ, UK.


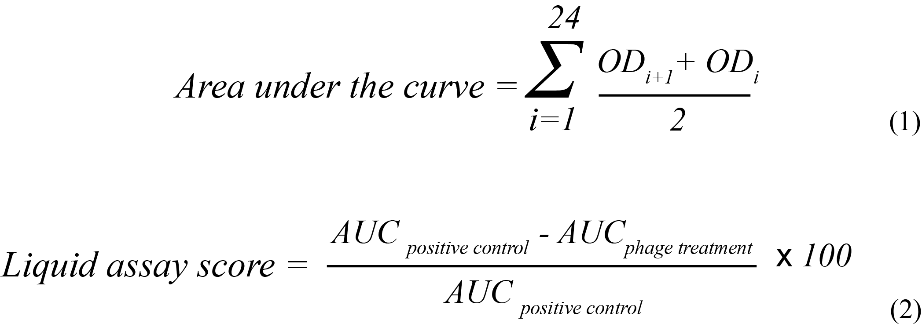


**Supplementary Equation 1 and 2.** Equations used for the calculation of Liquid assay score (LAS) for bacteriophages.


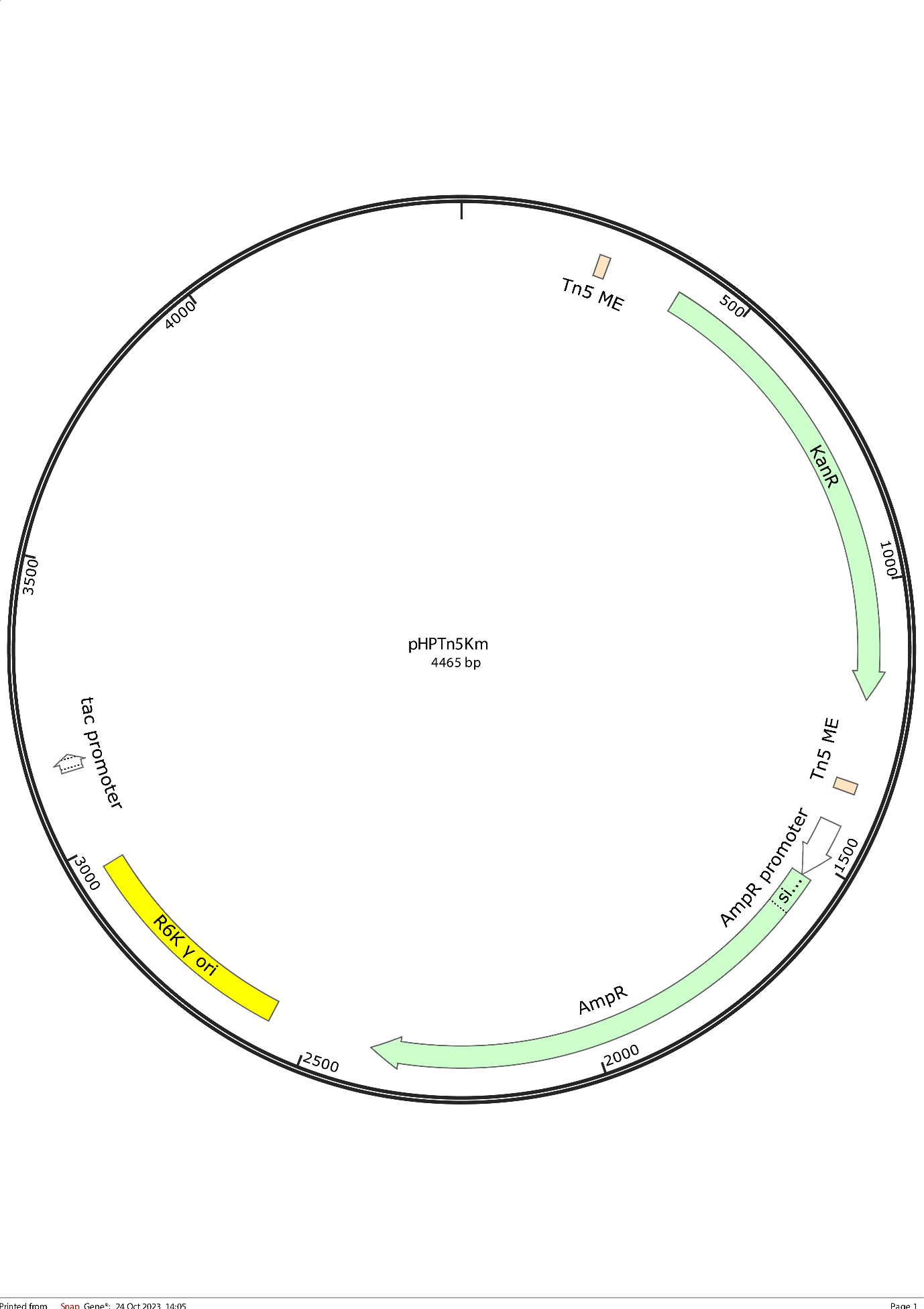


**Supplementary Figure 1. Genetic map of** plasmid pHPtn5Km. This plasmid was used to PCR amplify the Tn5::Km for preparation of transposomes used in the construction of a saturating transposon insertion mutant library.

**Supplementary Figure 2. Relationship of SPLA phages in the context of viral proteomes from phages within the Viral-Host Database.** The dendogram and host group was generated using proteome data with VipTree and Viral Family was annotated using genomes from Inphared. SPLA Salmonella phage isolates coloured with circles for viral genera *Berlinvirus* (red), *Seoulvirus* (orange), *Phikzvirus* (yellow), Tequintavirus (green), *Seunavirus* (Lightblue), *Rosemountvirus* (Purple) and *Lederbergvirus* (darkblue). Icons indicate predicted phage morphology.

**
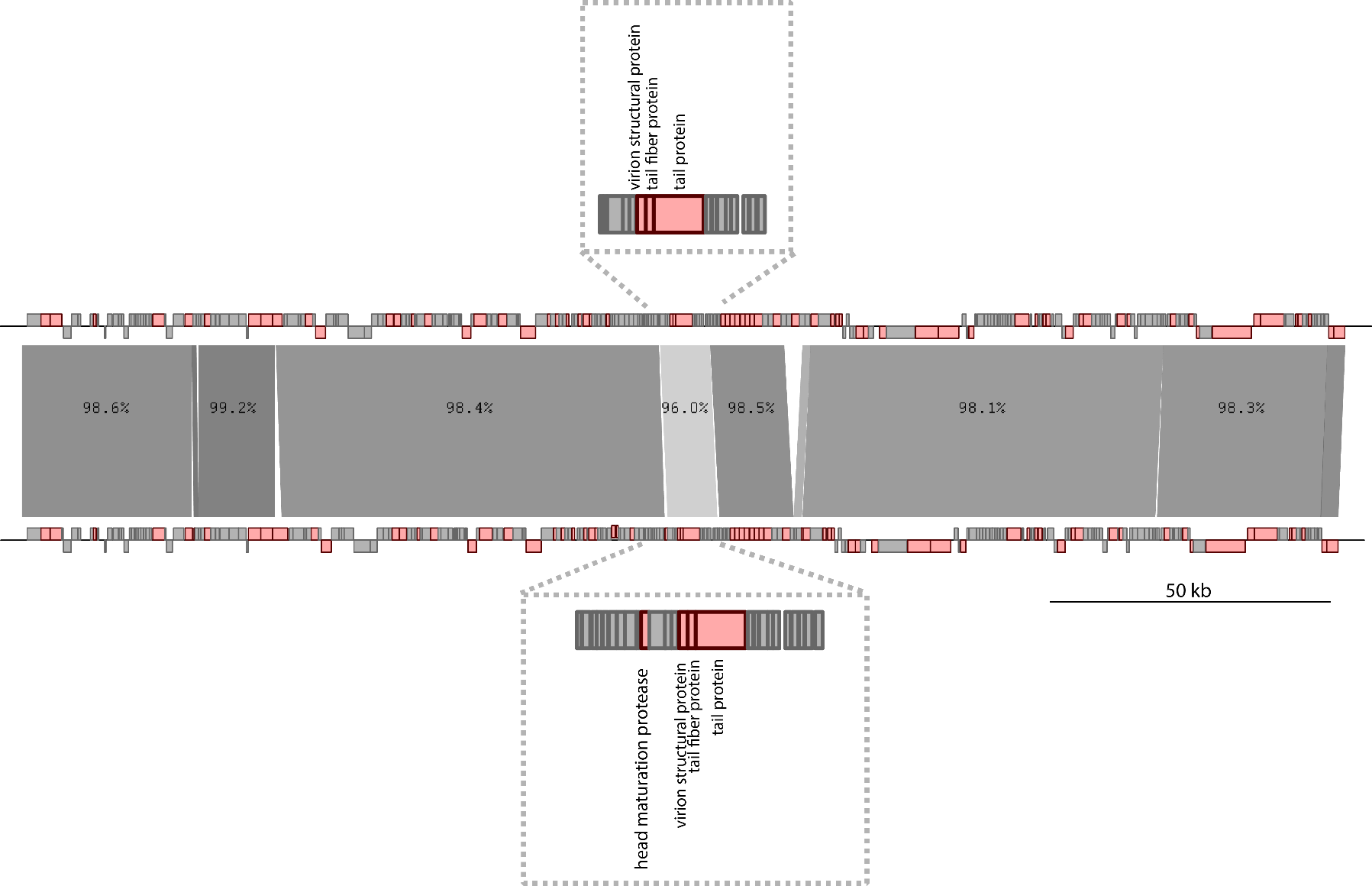
Supplementary figure 3.** Whole genome alignment of Seoulviruses SPLA1a and SPLA5c with nucleotide sequence identity and predicted function annotated using PHROGS software. The intensity of the shading indicates the nucleotide sequence identity. Hypothetical proteins are displayed in grey.

**Supplementary figure 4.** Nucleotide sequence alignment of *pgm* region of ST4/74 WT and ST4/74 SPL1a escape mutant (ST4/74*)

**Supplementary Tables available for download**

**Supplementary Table 1.** Bacterial strain collection used in the study for the isolation of bacteriophages and the investigation of bacteriophage host range.

**Supplementary Table 2.** List of Primer sequences used in the study.

**Supplementary Table 3.** List of log fold changes and statistical significance of transposon insertions within genes of Salmonella Typhimurium strain ST4/74 following exposure to six SPLA bacteriophages (SPLA1a, SPLA1b, SPLA2, SPLA5b, SPLA5c and SPLA11).
